# Thrombotic thrombocytopenic purpura (TTP)-like syndrome in the HIV era

**DOI:** 10.1186/s12959-018-0189-x

**Published:** 2018-12-13

**Authors:** Susan Louw, Reenelle Gounden, Elizabeth Sarah Mayne

**Affiliations:** Department of Molecular Medicine and Haematology, Faculty of Health Sciences, University of Witwatersrand and National Health Laboratory Service, Office 3B20, 7 York Road, Parktown, Johannesburg, 2191 South Africa

## Abstract

**Background:**

The thrombotic microangiopathies (TMAs) is a heterogeneous group of relatively uncommon but serious disorders presenting with thrombocytopenia and microangiopathic haemolysis. Thrombotic thrombocytopenic purpura (TTP) is one of these microangiopathic processes. HIV infection is an acquired cause of TTP but the pathogenesis is poorly understood. HIV-associated TTP was previously described to be associated with advanced immunosuppression. The incidence of HIV-related TTP was expected to decline with access to anti-retroviral therapy (ART).

**Methods:**

We undertook an observational study of patients with a diagnosis of TTP admitted to our hospital (CMJAH). The patient demographics, laboratory test results and treatment outcomes were recorded.

**Results:**

Twenty-one patients were admitted with a diagnosis of TTP during the study period. All patients had schistocytes and severe thrombocytopaenia. The presenting symptoms were non-specific and renal dysfunction and neurological compromise were uncommon. 77% of the patients were HIV-infected and, in 7 patients, TTP was the index presentation. The remainder of the HIV infected patients were on ART and the majority were virologically suppressed. A significant female preponderance was present. Only 4 of the 21 patients tested HIV negative with a positive Coombs test in 2. All patients in this cohort received treatment with plasma exchange therapy for a median period of 12 days with a 96.5% survival rate. Neither the baseline laboratory features nor the degree of immunosuppression was predictive of the duration of therapy needed for remission.

**Conclusion:**

HIV-related TTP is still a cause of morbidity and the clinical presentation is heterogeneous which may present a diagnostic challenge in the absence of sensitive biomarkers. Early treatment with plasma exchange is effective but expensive and invasive.

## Introduction

The thrombotic microangiopathies (TMAs) consist of a heterogeneous group of relatively uncommon but serious disorders presenting with thrombocytopenia and microangiopathic haemolysis with resultant characteristic red cell fragments on peripheral smear morphology. The pathophysiological disorders manifesting as TMAs include thrombotic thrombocytopenic purura (TTP), haemolytic uraemic syndrome (HUS), disseminated intravascular coagulopathy (DIC) and malignant hypertension [1–3].

Thrombotic thrombocytopenic purpura (TTP) is a microangiopathic thrombotic process which can result in multi-organ failure [4] characterised by widespread microvascular thrombi consisting of platelets and von Willebrand Factor (VWF). Thrombosis in TTP is initiated when haemostatically-active ultralarge VWF multimers accumulate in the circulation because of a relative or absolute deficiency of the cleaving protease, ADAMTS-13 (a disintegrin and metalloprotease with thrombospondin type 1 repeats, member 13) [5]. The sole function of ADAMTS-13 is to cleave VWF [4, 5]. Unravelling of VWF multimers in the high shear stress microvasculature results in spontaneous formation of platelet aggregates in organs such as the kidneys, heart and brain [4, 6]. TTP is reported as rare with an annual incidence of 1 per 1,000,000 of the population [7] but it carries a high mortality rate (10–20%) [1, 8]. Approximately 5% of cases are caused by a congenital deficiency of ADAMTS-13 [4]. The remainder (over 90%) are ascribed to auto-antibody formation against ADAMTS-13 arising spontaneously or secondary to a number of states including collagen vascular disorders like systemic lupus erythematosus (SLE), pregnancy, post-transplantation or after drug exposure [4, 5]. Female patients of African ancestry reportedly have the highest prevalence of acquired TTP often in the context of active SLE [4].

The diagnosis of TTP is based on clinical suspicion with supporting laboratory test results [9]. The diagnostic pentad consists of anaemia, neurological symptoms, fever, thrombocytopaenia and renal dysfunction although all 5 features are seen in fewer than 10% of patients [10]. Critical organ ischaemia can also present as gastrointestinal symptoms (in 35% of patients) and cardiac symptoms (25%) [10]. The laboratory tests reveal a severe bicytopaenia (anaemia and thrombocytopaenia) which is present in almost all cases with schistocytes (red cell fragments) on the peripheral smear and an elevated red cell distribution width (RDW). Laboratory features of haemolysis are present, notably a marked increase in lactate dehydrogenase (LDH) levels [4, 5, 11].

Early recognition and treatment is critical to prevent morbidity and mortality. Standard therapy is plasma exchange to supplement ADAMTS-13 and to remove ultra-large VWF multimers. Where plasma exchange is not readily available, plasma infusions can be performed [5, 12, 13].

HIV confers an increased risk for acquired TTP with a 15–40 fold higher incidence in this patient population compared with the HIV-uninfected population [14] but the pathogenesis is poorly understood [15]. TTP is reported to be more common in HIV-infected patients with advanced disease, low CD4^+^ T cell counts and with comorbidities (including Kaposi sarcoma and cryptococcal meningitis) [15–17]. Published case reports have documented that almost 100% of patients with acquired TTP have severe (< 10%) underlying ADAMTS-13 deficiency but levels of this protease in HIV-infected patients with TTP are variable and may be relatively preserved [18]. The incidence of HIV-related TTP was expected to decline with widespread access to antiretroviral therapy (ART) [10, 12–14] but evidence suggests that HIV is still an important cause of secondary TTP [4, 16].

The haematology ward at Charlotte Maxeke Johannesburg Academic Hospital (CMJAH), South Africa, has not experienced the predicted decline in HIV-related TTP or TTP-like syndrome [19] despite increased access to antiretroviral therapy. We decided to undertake a study to document the clinical presentation and treatment outcomes in these patients.

## Methodology

This was a retrospective observational study of all patients with a diagnosis of TTP or TTP-like syndrome admitted to the haematology ward at CMJAH, a 1000 bed, tertiary care academic hospital in Johannesburg, South Africa, over a period of 24 months. Patient demographic and clinical parameters, treatment and outcome were collated using a database. As this study was retrospective, informed consent could not be obtained. Ethics approval for the study was granted by the Human Research Ethics Committee (Medical) of the University of the Witwatersrand (Clearance Certificate No. M160134).

Descriptive statistics were derived for all parameters. For continuous variables, medians and interquartile ranges were derived. Where appropriate, a Mann-Whitney U test was performed and a *p* value of < 0.05 was considered statistically significant.

## Results

Twenty-one patients were admitted to CMJAH with a diagnosis of TTP during the study period. This hospital serves as a referral centre for healthcare facilities in Gauteng, the smallest but most densely populated province of South Africa. The hospital operates various specialist units, including haematology and oncology.

The patient demographics, laboratory test results and treatment are detailed in Table 1.

**Table 1:** TTP-like patient demographics, laboratory test results and treatment

	Age	Sex	Hb	WCC	Plts	RDW	Fragments	INR	PTT	D-dimers	DATT	LDH	Haptoglobin	Urea	Creatinine	HIV-infected	HIV VL	CD4+ T cell count	ARV therapy	Days of PE
Units	Yrs		g/dL	×10^9^/L	×10^9^/L	%			s	mg/L			g/L	mmol/L	ug/L		copies/mL	Cells/ul		
Reference range			11,6 16,4	3,9 12,60	186–454	12,4 17,3	Absent		31–48	< 0.25	Negative	100–190	0,4-2,4	2.1–7.1	49–90			332–1642		
1	48	M	8.8 (11.5)	10.4	33 (276)	33.8	3+	1.02	ND	1.48	ND	2365 (300)	< 0.1	9.9	126	Yes	LTDL	141	Yes	20
2	37	F	10.5 (12.4)	12.85	18 (351)	19.6	2+	1.81	32.6	ND	Neg	1073 (320)	< 0.1	12.4	105	Yes	LTDL	346	Yes	20
3	39	M	7.1 (12.9)	5.42	12 (268)	24.5	2+	1.09	32.6	ND	Neg	698 (468)	< 0.1	4	88	Yes	321,158	194	Yes	10
4	47	F	7.2 (12.8)	7.84	8 (219)	23.3	3+	1.14	30.8	3.20	ND	2023 (311)	< 0.1	9	128	Yes	191,000	134	No	25
5	32	F	4.6 (11.5)	8.56	8 (272)	30.5	3+	1.12	32.9	1.16	ND	941 (191)	< 0.1	1.8	57	Yes	373,000	80	No	11
6	27	F	8 (14.1)	4.92	35 (244)	32.6	3+	1.01	ND	11.90	Negative	2102 (180)	< 0.1	4.7	52	Yes	8642	182	Yes	10
7	43	F	10.2 (12.8)	8.4	16 (86)	24.6	3+	1.01	30.0	0.75	IgG +	2005 (160)	< 0.1	9.1	79	No	N/A	N/A	N/A	12
8	30	F	7.3 (9.7)	6.56	15 (175)	33.9	3+	1.15	ND	11.90	Negative	1836 (178)	ND	4.8	60	Yes	42	113	Yes	10
9	35	F	5.5 (11.1)	9.17	5 (280)	26.6	3+	1.2	31.0	6.49	Negative	1768 (182)	< 0.1	3.8	71	Yes	ND	585	Yes	18
10	45	F	6.7 (10.4)	4.16	18 (105)	22.7	3+	1.06	44.3	ND	IgG +	950 (227)	< 0.1	4.1	67	Yes	228,912	54	No	20
11	43	F	7.8 (9.5)	10.4	18 (23)	23.9	1+	1.62	43.1	0.56	IgG +	1441	ND	17	211	No	N/A	N/A	N/A	12^b^
12	44	F	6.3 (14.3)	13.91	3 (290)	27.9	3+	1.21	43.1	10.00	ND	2708 (478)	< 0.1	7.8	113	Yes	2150	187	Yes	18
13	43	F	6.1 (10.4)	10.5	10 (274)	25	3+	1.17	36.9	7.13	ND	1357 [32]	< 0.1	5.5	94	Yes	72,905	264	No	12
14	40	M	7.4 (10.8)	4.15	21 (254)	20.6	2+	1.11	41.1	1.0	IgG +	1068 (273)	< 0.1	7.7	101	Yes	26,065	215	No	14
15	34	F	6.5 (10.7)	8.96	6 (270)	19	2+	1.35	33.8	17.00	Negative	1683 (257)	< 0.1	6.4	96	No	N/A	N/A	N/A	12
16	56	F	5.8 (8.5)	14.7	5 (328)	34.2	3+	1.3	33.5	ND	Negative	3339 (305)	< 0.1	6.6	80	Yes	LTDL	180	Yes	11
17	34	F	6.7 (12.5)	13.67	5 (248)	27.2	3+	1.28	31.7	0.00	IgG +	2404 (206)	< 0.1	6.5	110	Yes	562,635	421	No	11
18	44	M	7.5	19.35	25	20.2	3+	1.14	34.0	ND	ND	5472	ND	27.3	262	Unknown	ND	ND	Unknown	1^a^
19	33	F	6.5 (10.2)	8.9	6 (203)	19	2+	1.35	33.8	17.00	Negative	1683 (195)	< 0.1	6.4	96	No	N/A	NA	N/A	12
20	38	F	7.9 (9.5)	5.6	3 (253)	15.2	2+	1.05	43.6	1.93	IgG +	827 (244)	< 0.1	5.3	67	Yes	2,030,000	100	No	15
21	35	M	2.7 (7)	13.2	29 (134)	13.5	1+	2.38	92.3	0.97	IgG+	978 (476)	< 0.1	5.6	136	Yes	1,010,000	34	No	15
Median	39		7.1 (10.95)	9.065	12 (253.5)	24.5		1.14	33.6	2.565		1683 (244)		6.6	96		209,956	181		12
IQR (25–75%)	34–44		6,35-7725	6,56-13,2		20,2 27,725	6–20,3	1.08–1.2	32–43	0.99–10.5		1069-2299		1.2–3	73–122.7		21,709-420,409	110–227		11–18

^a^Patient demised on day 1. ^b^ Patient defaulted after 12 days of plasma exchange. Values in brackets denote discharge haemoglobin, platelet and LDH levels

Abbreviations: *Yrs* years, *Hb* haemoglobin, *WCC* White cell count, *RDW* red cell distribution width, *Plts* platelets, *LDH* lactate dehydrogenase, *ARV* antiretroviral therapy, *VL* viral load, *LTDL* viral load lower than detectable limit

*PE*:plasma exchange, *N/A* not applicable

All patients presented with laboratory features of a microangiopathic thrombotic process with high numbers of schistocytes on the peripheral smear and a severe evolving thrombocytopaenia (median platelet count at diagnosis of 12 × 10^9^/L). The majority of patients were transferred to our tertiary care facility from other clinical sites. The presenting symptoms were non-specific commonly including headache, lethargy and gastrointestinal symptoms (diffuse abdominal pain with mild diarrhoea). Only 1 patient had laboratory evidence of renal dysfunction (urea of 27.3 mmol/L (normal reference range 2.1–7.1 mmol/L) and a creatinine of 262 μmol/L (normal reference range 49–90 μmol/L)) together with confusion. This patient demised shortly after admission and could not be further investigated. Only 1 patient presented with active bleeding (ecchymoses).

Only 4 of the 21 patients tested HIV negative. All of these patients were female and were investigated extensively for underlying autoimmune disease. A positive Coombs test for IgG antibodies was seen in 2 of these patients. One patient could not be tested for HIV infection. The remaining 17 patients were HIV-infected and in 7 (47%), TTP was the initial presentation of the underlying HIV infection. These patients were untreated and had a median viral load of 228,912 (interquartile range: 131952.5–467,817.5 copies per ml) and a median CD4^+^ T cell count of 134 cells/uL (interquartile range: 90–305 cells/uL). The HIV infected ART naïve patients were all commenced on ART with as a single, fixed-dose combination (FDC) tablet containing tenofovir (TDF), emtricitabine (FTC) and efavirenz (EFV) following appropriate pre- and post-test counselling. The remainder of the infected patients were on ART and the majority were virologically suppressed with only 2 patients having detectable viral loads. These patients did, however display a wide range of CD4^+^ T cell counts with a median value of 184.5 cells/mm^3^ and an interquartile range of 170–232 cells/uL. There was a significant female preponderance in the HIV-infected patients with only 4 of these patients (23.5%) being male. Male patients presented with significantly higher platelet counts than females (median of 16 × 10^9^/l vs 6 × 10^9^/l, *p* < 0.004). No other significant differences between male and female patients was noted with respect to degree of anaemia, levels of LDH or duration of plasma exchange.

Haptoglobin was analysed in the 19 of the 21 patients and was consistently reduced to < 0.01 g/L (normal reference range 0.4–2.4 g/L) despite preserved liver synthetic function as indicated by normal albumin concentration.

All patients in this cohort received treatment with plasma exchange therapy for a median period of 12 days (range 1–20 days) with a 96.5% survival rate (one patient demised 24 h post-admission). One patient refused hospital care after 12 days of treatment. The remaining patients were discharged post-exchange therapy to out-patient follow-up care after their platelet counts had normalised and had remained stable for 2 days and their LDH levels had declined to normal [5, 11, 12]. Neither the baseline platelet counts, LDH level nor the degree of immunosuppression was predictive of the duration of plasma exchange needed for remission.

## Discussion

Although TTP incidence was reported to be declining in HIV-infected patients with increased access to ART [4], this has not been the experience at our centre. Over a 24-month period, we admitted 21 patients with a diagnosis of TTP-like syndrome. The majority (75%) of patients admitted to CMJAH with a diagnosis of TTP were HIV-infected in keeping with previous studies at our own and other centres in South Africa [13, 15, 16, 18].

The clinical presentation in our cohort was heterogeneous with none of the patients displaying the classical diagnostic pentad [15]. Of note, only 1 patient had objective laboratory features of renal dysfunction and no patient had clear evidence of neurological dysfunction. Consistent features in our presenting cohort were severe evolving thrombocytopaenia and haemolytic anaemia with numerous schistocytes on the peripheral smear.

Applying the PLASMIC score [2, 20] consisting of the presence of thrombocytopenia and haemolysis with reduced mean red cell volume (MCV), preserved renal function and absence of underlying malignancy with no history of receiving a tissue transplant, to the patients in our cohort (Table 1) would have changed the diagnosis of TTP to another TMA for patients 1, 11 and 21. The PLASMIC score was however developed to identify patients with TMA and severe ADAMTS-13 deficiency manifesting as TTP. Previous studies at our centre have demonstrated that a significant proportion of patients with HIV-associated TTP-like syndrome do not have anti-ADAMTS-13 inhibiting antibodies [18]. Other studies have shown heterogeneity in levels of ADAMTS-13 in these patients ranging from very severe deficiency (< 5%) in up to 44% of patients to normal levels in up to 30% of patients and factors other than ADAMTS-13 deficiency are therefore postulated as pathogenic mechanisms including endothelial injury by HIV itself [21–25], damage by other opportunistic infections or endothelial activation caused by HIV-associated chronic inflammation [15, 18, 26]. This endothelial injury is postulated to results in release of stored VWF which overwhelms the capacity of ADAMTS-13 culminating in a consumptive deficiency. A similar transient deficiency in ADAMTS-13 activity can be seen in healthy volunteers after DDAVP administration which results in release of VWF from endothelial cells [27]. The application of scoring systems such as the PLASMIC score in our environment is currently inappropriate given the high mortality if definitive treatment is not promptly instituted.

Endothelial damage and local activation of coagulation probably also results in an isolated, elevated D-dimer level in these patients [18, 28]. The patients in this study had consistently raised D-dimer levels with a median level of 2.565 mg/L although other coagulation parameters were not deranged. The inconsistency between the decrease in ADAMTS-13 levels and presence of inhibiting antibodies may explain the therapeutic effect of plasma infusion without exchange therapy in HIV-related TTP [13]. The elevated D-dimer levels in the 4 HIV-uninfected patients in the current cohort may relate to sub-clinical bleeding in view of the significant thrombocytopaenia. A differential diagnosis of Evan’s syndrome (autoimmune haemolytic anaemia with thrombocytopaenia) was considered in 2 of the HIV-uninfected patients although the laboratory features were non-diagnostic (specifically the Coombs testing was equivocal). The therapy for TTP is, however, effective for patients with Evan’s syndrome and both patients responded well.

HIV-associated TTP or TTP-like syndrome is prevalent in our centre despite increased access to anti-retroviral therapy (ART). The patients in this study did not show consistently low CD4^+^ T cell counts or high HIV viral loads. Other co-morbid diseases like Kaposi Sarcoma and cryptococcal meningitis were not overtly evident in the HIV-infected patients in our cohort. Previous studies have shown that TTP is seen in patients with profound immunosuppression and often with AIDS-defining conditions like Kaposi Sarcoma [5, 9, 14, 18, 28, 29]. 8 of the 15 HIV-infected patients in the current study were on ART at the time of admission with TTP. The HIV viral loads ranged from below detectable limit (in the majority of these patients) to 2,030,000 copies/ml. The viral load and CD4+ T cell count did not predict the duration of plasma exchange needed to achieve remission although the HIV-uninfected patients did achieve remission after on average fewer days of plasma exchange therapy. For 7 patients, TTP was the index presentation of their HIV infection.

The differential diagnosis for TTP includes other thrombotic microangiopathies (TMAs) importantly disseminated intravascular coagulopathy (DIC) and haemolytic uraemic syndrome (HUS) [30]. The diagnosis of TTP and TTP-like syndrome in our unit is made on the basis of severe thrombocytopaenia, elevated LDH levels and schistocytes on the peripheral smear in the correct clinical setting (including the presence of HIV infection). DIC is generally excluded when the coagulation parameters (with the exception of the D-dimers) are normal [28]. HUS would only be considered in patients with current or historical diarrhoea and severe renal dysfunction. In our cohort, only 1 patient presented with any significant renal dysfunction. The absence of a single highly sensitive and specific marker for TTP in HIV infection is a significant impediment to early diagnosis and care, since other TMAs cannot always be excluded with certainty. Of note, the Coombs test may be non-specifically positive in HIV-infected patients and the clinical and diagnostic significance of this is uncertain [31].

All 21 patients in this cohort received daily plasma exchange therapy with Fresh Frozen Plasma (FFP). In patients who responded slowly either the volume of exchange was increased to 1.5 plasma volumes and/ or cryo-poor plasma at the treating clinician’s discretion [32]. Steroids (prednisolone at 1 mg/kg) were prescribed to all HIV-uninfected patients in the cohort but were used inconsistently in the HIV-infected individuals [4, 5]. Only 1 patient demised during this study after 1 day of treatment. The cause of death in this patient was not clear. This patient presented with the highest LDH levels in the cohort (5472 U/L) suggesting severe haemolysis and tissue damage [4]. All other patients responded to therapy and were discharged with normal platelet counts and LDH levels. All HIV-infected patients who were not on ART were placed on first-line therapy consisting of single, fixed-dose combination (FDC) tablet containing tenofovir (TDF), emtricitabine (FTC) and efavirenz (EFV [32]. The 4 HIV-uninfected patients responded quicker to plasma exchange. A possible differential diagnosis of Evans syndrome (autoimmune haemolytic anaemia with thrombocytopaenia) was considered in these patients.

## Conclusion

TTP or TTP-like syndrome is a significant cause of morbidity in patients infected with HIV. There is no clear link to opportunistic infections in all cases or to severe levels of immunosuppression. In some cases, TTP represents the presenting complaint for patients with undiagnosed HIV infection. Early treatment with plasma exchange is highly effective but expensive and invasive (requiring insertion of large bore catheters). The absence of highly sensitive and specific biomarkers to diagnose TTP in this subset of patients is a challenge.

### Limitation of the study

Only 8 (less than 50%) patients in this cohort were on ART limiting the statistical ability to draw a conclusion with regard to the effectiveness of ART in preventing the development of TTP. In addition, ADAMTS-13 antibodies and levels are not routinely measured at our centre which makes the application of scoring systems like the PLASMIC score difficult. Previous studies [18] suggest, however, that this score may need modification in our patient population due to the heterogeneity in ADAMTS-13 levels.
